# Minimally invasive approaches to atrial septal defect closure

**DOI:** 10.1016/j.xjtc.2022.02.037

**Published:** 2022-04-02

**Authors:** Igor E. Konstantinov, Yasuhiro Kotani, Edward Buratto, Antonia Schulz, Yaroslav Ivanov

**Affiliations:** a Department of Cardiac Surgery, Royal Children's Hospital, Melbourne, Australia; b Department of Paediatrics, University of Melbourne, Melbourne, Australia; c Heart Research Group, Murdoch Children's Research Institute, Melbourne, Australia; d Melbourne Children's Centre for Cardiovascular Genomics and Regenerative Medicine, Melbourne, Australia; e Okayama University Graduate School of Medicine, Dentistry and Pharmaceutical Sciences and Okayama University Hospital, Okayama, Japan

**Keywords:** minimally invasive heart surgery, atrial septal defect, cosmetic surgery, children

**Figure:**
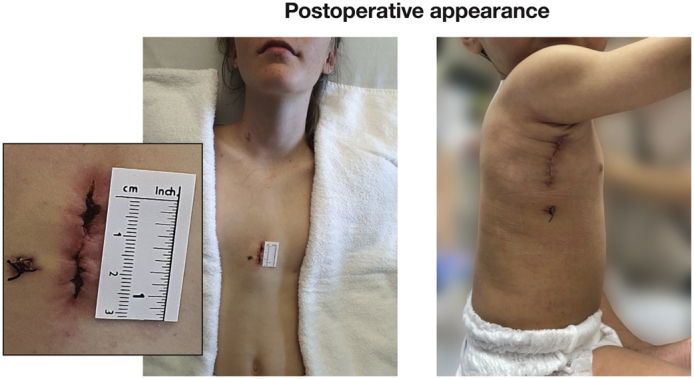
ASD closure via partial sternotomy in 42-kg and via right thoracotomy in 7.7-kg patient.

**Central Message**

Minimally invasive closure of atrial septal defects can be safely achieved with a range of techniques. Currently, partial sternotomy and right axillary thoracotomy are the most widely used approaches.

Percutaneous device closure is currently the preferred treatment for children with secundum atrial septal defects (ASDs).^1^ However, there is a group of patients who are not suitable for device closure, due to insufficient margins or the large size of the defect, in whom surgical closure is required. Furthermore, there is an evolving understanding of significant adverse reactions to septal occlusion devices due to nickel allergy. In some patients, surgical removal may be required to alleviate symptoms attributed to nickel allergy.^2^, ^3^, ^4^, ^5^ Although newer septal occlusion devices have been shown in vitro to have significantly lower nickel elution than the previously used devices,^5^ systemic allergic contact dermatitis to nickel has also been reported with these new devices.^6^ Given the ongoing need for surgical ASD closure in a significant proportion of patients, it would be reasonable to employ minimally invasive approaches to reduce surgical trauma and improve cosmesis.

As experience with minimally invasive approaches has increased in pediatric cardiac surgery, its application has been extended from “simple” lesions such as ASD^7^^,^^8^ to more complex lesions such as tetralogy of Fallot^9^, ^10^, ^11^ and mitral valve repair.^12^ There appears to be a consensus that minimally invasive repair of ASD is a reasonable and safe alternative to conventional sternotomy.^13^ The improved cosmetic result is clearly the major advantage of minimally invasive surgery. This must be achieved without increase in surgical risk. The minimally invasive approaches appear to provide similarly excellent results to conventional sternotomy^8^^,^^10^^,^^14^ with potential benefits of decreased length of hospitalization,^14^ postoperative pain, and hospital cost.^15^ In fact, it has been suggested that the minimally invasive approach should be adopted as a new “standard” for surgical ASD closure.^10^^,^^13^^,^^16^

A great number of minimally invasive approaches have been described, including partial sternotomy,^7^^,^^8^^,^^17^^,^^18^ transxiphoid approach,^19^^,^^20^ anterolateral,^16^^,^^21^^,^^22^ and posterolateral^23^^,^^24^ right-sided thoracotomy, right axillary approach,^12^, ^13^, ^14^^,^^25^, ^26^, ^27^ and video-assisted thoracoscopic surgery, albeit, the latter for adolescents and adults.^28^^,^^29^ These approaches may^28^ or may not require special instrumentation.^8^^,^^13^ Reproducibility, learning curve, and transfer of surgical skills to trainees are also important aspects of minimally invasive ASD closure. In the modern era, 2 approaches appear to have gained the most widespread adoption: right thoracotomy and partial median sternotomy.

## Right Thoracotomy

**Figure 1:**
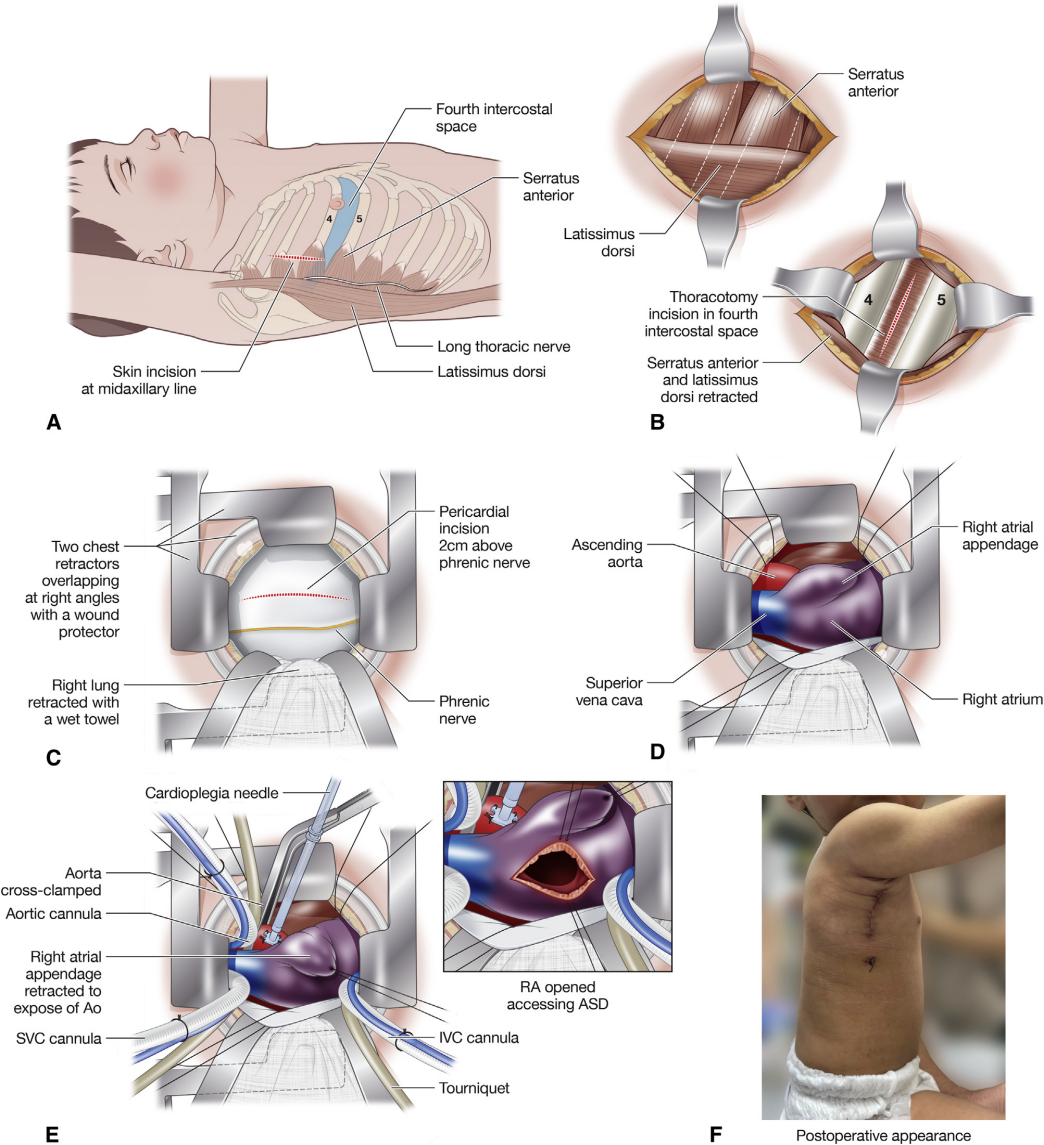
Minimally invasive right thoracotomy approach for atrial septal defect closure. A, Skin incision in relation to local topographical anatomy. B, Muscle-sparing approach is performed to enter the chest between the fourth and fifth ribs. C, Pericardium is opened anterior to the phrenic nerve. D, The heart is exposed. E, Cardiopulmonary bypass is established, aorta is crossclamped, cardioplegia is administered, and atrial septal defect is exposed. F. Cosmetic result in a 7.7-kg child. Informed consent to produce the patient's image was obtained from the parent.

Minimally invasive ASD closure through a midaxillary approach was initially reported by Schreiber and colleagues^13^ from Munich in response to unsatisfactory results from the right anterolateral thoracotomy.^30^ The midaxillary approach is appealing, as the area is least covered by chest wall muscles, is far away from the immature breast tissue, and provides a direct plane of vision to the atrial septum. Access may be achieved either through a transverse or vertical (Figure 1, *A*) midaxillary skin incision, allowing a muscle-sparing approach to the fourth intercostal space (Figure 1, *B*). A vertical incision is made in the pericardium and care is taken to avoid injury to the phrenic nerve (Figure 1, *C*). Direct vision of the aorta, superior vena cava, and right atrium is achieved with the use of soft-tissue retractors (Figure 1, *D*).

In the majority of cases, it is possible to achieve aortic cannulation directly via the thoracotomy (Figure 1, *E*). However, when difficulties in cannulation occur via this approach, they can be difficult to manage due to the limited space and access.^31^ As such, surgeons need to be prepared for alternative sites of arterial cannulation, such as the femoral artery. However, a body weight of less than 10 to 15 kg^12^^,^^32^ is generally considered to be a relative contraindication to femoral artery cannulation. The vertical axillary incision is hidden by the adducted arm, providing excellent cosmesis (Figure 1, *F*).

Myocardial protection may be achieved by either fibrillatory arrest or aortic crossclamping and cardioplegic arrest. Some institutions prefer fibrillatory arrest, however, it is crucial that the surgeon is extremely vigilant in ensuring that the fibrillation pads constantly maintain contact with myocardium and that the fibrillatory arrest is continuously assessed by electro and echocardiography.^12^ Inadvertent defibrillation and ejection of air can result in massive air embolism and catastrophic neurologic complications.^12^ This can be avoided by aortic crossclamping and cardioplegic arrest, which can be achieved directly via the thoracotomy (Figure 1, *E*). The result of surgical ASD closure must be nothing but perfect. Thus, we would always perform aortic crossclamping to eliminate any risk of air embolization. This technique has been used in 101 children in Okayama University Hospital without any mortality, morbidity or conversion to full thoracotomy.

Schreiber and colleagues^13^ reported 36 patients who had minimally invasive ASD closure through the right midaxillary approach with excellent cosmetic outcomes. Nevertheless, they recommended restricting the approach to patients older than 3 or 4 years.^13^ Another series by Dave and colleagues^12^ demonstrated that the midaxillary approach could be used not only for ASD closure, but also be expanded to more complex operations. Interestingly, in their series the youngest of their 62 patients undergoing ASD closure was 4.5 months and the minimum weight was 3.8 kg. Since then, there have been a number of reports of midaxillary approach for the minimally invasive ASD closure,^10^^,^^14^^,^^27^ including one large series of 244 consecutive patients.^10^

While similar access can be achieved via a right anterolateral thoracotomy,^16^^,^^21^^,^^22^ some cosmetically undesirable outcomes have been reported.^30^^,^^33^ Impaired breast development is of concern, as it is challenging to determine the appropriate length and position of the incision in a child with respect to the immature breast tissue. According to Bleiziffer and colleagues^30^ right breast asymmetry was reported in 61% of female patients who underwent ASD closure via right anterolateral thoracotomy before onset of puberty compared with no such events in standard full median sternotomy group. As a balancing argument, they reported that 76% of patients in the thoracotomy group perceived their cosmetic results as excellent in contrast to 39% of patients in standard full median sternotomy group. Similarly, Isik and colleagues^33^ reported breast asymmetry occurrence in 60% and mild sensory deficit in the mammary area in 16% of women who underwent ASD closure in prepubertal age via anterolateral thoracotomy. Clearly, the anterolateral thoracotomy approach should be used cautiously, if at all, in prepubertal female patients.

## Partial Sternotomy

**Figure 2:**
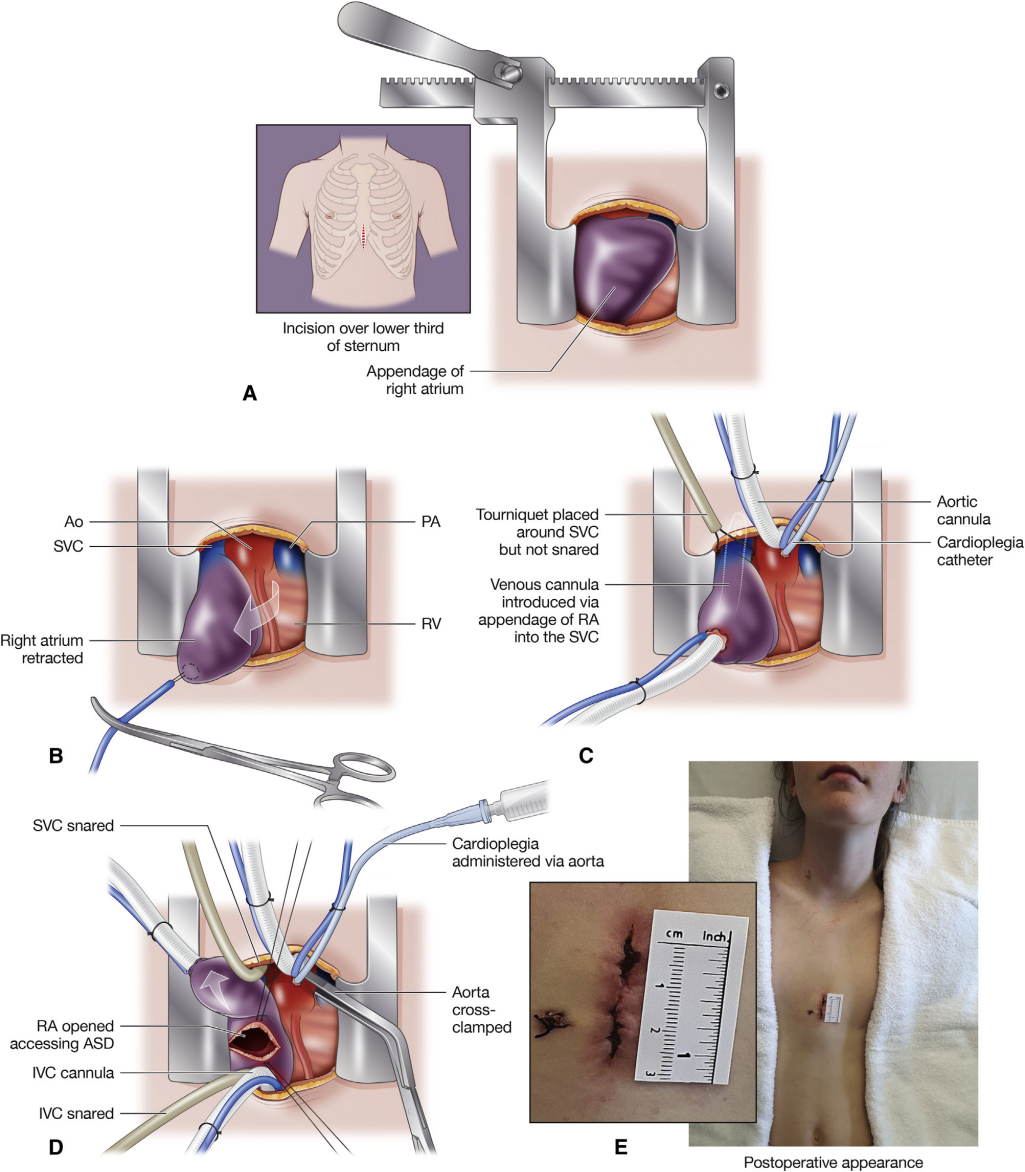
Minimally invasive lower sternotomy approach for atrial septal defect closure. A, Skin incision over the lower part of the sternum, exposing the heart. B, Purse-string is placed on the appendage of the right atrium to facilitate aortic exposure. C, Cardiopulmonary bypass is instituted. D, The inferior vena cava is cannulated, aorta is crossclamped, cardioplegia is administered, and atrial septal defect is exposed. E, Cosmetic result in 42-kg girl. Informed consent to produce the patient's image was obtained from the parent. *Ao*, Aorta; *PA*, pulmonary artery; *SVC*, superior vena cava; *RV*, right ventricle; *RA*, right atrium; *ASD*, atrial septal defect; *IVC*, inferior vena cava.

A limited midline sternotomy incision is an alternative minimally invasive approach for ASD closure. The patient is positioned and draped as for conventional midline sternotomy. A limited skin incision is placed over the inferior third of the sternum, and a limited sternotomy is performed (Figure 2, *A*).

Cannulation is achieved directly and facilitated by initial placement of a right atrial purse-string suture to retract the right atrial appendage and expose the aorta (Figure 2, *B*). Superior vena cava cannulation can be simplified by using a malleable cannula inserted via the right atrial appendage (Figure 2, *C*). Standard placement of the inferior vena cava and cardioplegia cannulae can be achieved on cardiopulmonary bypass (Figure 2, *D*). A conventional right atriotomy is performed, allowing the ASD to be closed. With experience, the length of the incision can be decreased to only 3 to 4 cm (Figure 2, *E*). This technique has been performed in the Royal Children's Hospital in Melbourne in 77 children without any mortality, morbidity, or conversion to full sternotomy as previously reported.^8^

Advantages of the partial sternotomy may include short learning curve,^8^ and same,^8^ or very similar, surgical equipment^7^^,^^18^ used for conventional full median sternotomy. Most importantly, there is the advantage of rapid conversion to full median sternotomy if required; however, published series from Boston^7^ and Melbourne^8^ have demonstrated that such conversion was not required.

Importantly, no increase in operative or postoperative morbidity has been reported with ministernotomy approaches.^7^^,^^8^ Anecdotally, a greater incidence of pericardial effusion in ministernotomy group was observed; therefore, routine creation of pericardial window has been recommended.^7^ Interestingly, although it was hoped that minimally invasive surgery would result in faster postoperative recovery, this has not been observed in series reporting the results of ministernotomy ASD closure.^7^^,^^8^^,^^18^

## Alternative Approaches

Although right thoracotomy and partial sternotomy are the most widely used approaches, alternative techniques have also been reported. Several groups have reported trans-xiphoid approach.^19^^,^^20^^,^^34^ However, Hagl and colleagues^34^ found that it compromised exposure of the ascending aorta, resulting in difficulties with crossclamping, administration of cardioplegia, and especially deairing. Perhaps, the difficulties with direct aortic cannulation may be alleviated with femoral vessels cannulation; however, this approach may not be feasible in smaller patients.^32^ Although the transxiphoid approach may provide excellent cosmetic outcomes, it appears to introduce considerable technical complexity.

Video-assisted thoracoscopic ASD closure allows the surgeon to achieve anatomical visualization without excessive tissue traction and extended incisions. While its safety and efficacy has been demonstrated in a large group of adult patients,^29^ the experience in the pediatric patients seems to be limited.^28^^,^^35^ Although Wang and colleagues^28^ demonstrated the feasibility and safety of thoracoscopic surgical ASD closure in 26 children weighing 13.5 to 22 kg, they also highlighted that this type of surgery required meticulous surgical technique with careful surgical planning. Furthermore, crossclamp times are much longer in the thoracoscopic group compared with midaxillary access group, which reflects additional complexity of this surgery.^14^ In the end, it leaves the patient with 3 port incisions on the right chest wall.^14^ Again, the need for femoral arterial cannulation imposes limitations on the size of patients who are suitable for this approach.^28^

Irrespective of the approach chosen (Table 1), minimally invasive ASD closure should fulfill several criteria. Most importantly, the safety must be equivalent to the traditional full sternotomy approach.^36^ In ASD closure, any result short of perfection is unacceptable due to the high standards of safety set by device closure and conventional surgical approach. Learning curve, the need for additional training, and equipment are important factors when implementing a minimally invasive ASD program. Finally, the cosmetic result should be considered for each patient individually, and this includes the prominence of the location, the length of the incision and finally the impact on developing breast tissue.

**Table 1:** Summary of the literature on minimally invasive ASD closure in children

Author	Years	Number	Age range	Weight range	Approach	Myocardial protection	Cannulation strategy	Defects
**Thoracotomy**								
Yoshimura et al, 2001^23^	1983-2000	126	1-15 y	6.9-56 kg	Posterolateral thoracotomy	Fibrillatory arrest	Central	ASD
Liu et al, 2000^11^	1994-1999	683	4 mo to 7 y	5-40 kg	Right thoracotomy	Crossclamp and cardioplegia	Central	ASD (403), ToF (65), pAVSD (16, VSD (24), MV repair (4), cor triatriatum (2) LVOTO (2), PS (2). LA myxoma (1), LCA to LV fistula (1)
Formigari et al, 2001^15^	1996-1998	71	Median 5.1	Median 20.5 kg	Right anterolateral thoracotomy	Crossclamp and cardioplegia	Central	ASD
Giamberti et al, 2000^9^	1997-1999	100	17 mo to 16 y	9-65 kg	Submammary thoracotomy	Crossclamp and cardioplegia	Central	ASD (78), VSD (7), ToF (6), pAVSD (5), DCRV (2), Fontan (1).
Vida et al, 2013^16^	1998-2013	141	8 mo to 12 y	7-45 kg	Right anterolateral thoracotomy	Fibrillatory arrest	Peripheral	ASD
Dave et al, 2009^12^	2001-2007	123	0.4-19.4 y	3.8-62 kg	Right axillary thoracotomy	Fibrillatory arrest	Mostly peripheral	ASD (84), pAVSD (19), and VSD (20)
Mishaly et al, 2008^21^	2002-2007	75	1.2-56 y	8.5-118 kg	Anterior thoracotomy	Fibrillatory arrest	Peripheral	ASD (37), pAVSD (11), VSD (4), DCRV (1), MV repair (8), PAPVD (14)
Schreiber et al, 2005^13^	2003-2004	36	4-14 y	15-69 kg	Right axillary thoracotomy	Fibrillatory arrest	Central	ASD
Yan et al, 2013^27^	2003-2010	52	0.8-34.9 y	9-63 kg	Vertical axillary thoracotomy	Crossclamp and cardioplegia	Central	ASD (20), VSD (26), pAVSD (6)
**Mini-sternotomy**								
Black and Freedom, 1998^17^	1995-1996	23	19 mo to 15 y	11-62 kg	Mini-sternotomy	Crossclamp and cardioplegia in majority	Central	ASD
Bichell et al, 2000^7^	1996-1998	135	6 mo to 25 y	Not reported	Mini-sternotomy	Crossclamp and cardioplegia	Mostly central	ASD
Sebastian et al, 2009^18^	2004-2007	79	1 mo to 10 y	3.5-40 kg	Mini-sternotomy	Crossclamp and cardioplegia	Central	ASD (34), pAVSD (3), TAPVD (1), PV plasty (1), VSD (40)
Konstantinov and Buratto, 2021^8^	2010-2020	55	6 mo to 16 y	Mean 22.8 kg	Mini-sternotomy	Crossclamp and cardioplegia	Central	ASD
**Alternative approaches**								
Barbero-Marcial et al, 1998^19^	1996-1997	10	6 mo to 14 y	Not reported	Transxiphoid	Crossclamp and cardioplegia	Peripheral	ASD
Van de Wal, 1998^20^	1996-1997	26	6 mo to 14 y	Not reported	Transxiphoid	Crossclamp and cardioplegia	Both central and peripheral	ASD
Hagl et al, 2001^34^	1997-1998	5	4 mo to 10 y	Not reported	Transxiphoid	Crossclamp and cardioplegia	Central	ASD
Wang et al, 2011^28^	2009-2010	28	4.5-8 y	13.5-22 kg	Thoracoscopic	Crossclamp and cardioplegia	Peripheral	ASD

*ASD*, Atrial septal defect; *TOF*, tetralogy of Fallot; *pAVSD*, partial atrioventricular septal defect; *VSD*, ventricular septal defect; *MV*, mitral valve; *LVOTO*, left ventricular outflow tract obstruction; *PS*, pulmonary stenosis; *LA*, left atrium; *LCA*, left coronary artery; *LV*, left ventricle; *DCRV*, double-chambered right ventricle; *PAPVD*, partial anomalous pulmonary venous drainage; *TAPVD*, total anomalous pulmonary venous drainage; *PV*, pulmonary valve.

## Conclusions

A range of techniques can be used to achieve a cosmetic approach to ASD closure. In particular, partial sternotomy and midaxillary thoracotomy appear be the most widely adopted techniques, providing excellent cosmesis, allowing conventional approaches to bypass and myocardial protection as well as achieving outcomes with safety equivalent to traditional median sternotomy.

### Conflict of Interest Statement

The authors reported no conflicts of interest.

The Journal policy requires editors and reviewers to disclose conflicts of interest and to decline handling or reviewing manuscripts for which they may have a conflict of interest. The editors and reviewers of this article have no conflicts of interest.
